# Lupus. Biniodide of Mercury. Dippel’s Animal Oil

**Published:** 1854-12

**Authors:** 


LUPUS. BINIODIDE OF MERCURY. DIPPEL'S ANIMAL OIL.—Cazenave employs as a local application a strong ointment, composed of equal parts of the biniodide of mercury and of a mixture of lard and almond oil. In winter the proportion of oil is increased, in order that the ointment may be liquid, and may spread easily over the skin. The ointment produces intense congestion and redness of the skin, and severe pain. If the parts are covered with cuticle, vesications and pustules are produced, which crust over. If the parts are ulcerated, a thick albuminous secretion is poured out, which forms a crust. When the crusts are detached in five or six days, the lupose tubercles are found to be much reduced in size. Two or three applications of the ointment, at intervals of a week, are sufficient to reduce them to the level of the skin. No unfavorable symptom has been known to be produced by the absorption of the biniodide.

Cazenave also sometimes employs as a local application, Dippel's animal oil, in the place of the biniodide; and these two remedies constitute the only local measures. The cod liver oil is employed internally.—*Bull. Gén. de Thér.*

